# Genetic Characterization of *Listeria* from Food of Non-Animal Origin Products and from Producing and Processing Companies in Bavaria, Germany

**DOI:** 10.3390/foods12061120

**Published:** 2023-03-07

**Authors:** Simone Wartha, Nancy Bretschneider, Alexandra Dangel, Bernhard Hobmaier, Stefan Hörmansdorfer, Ingrid Huber, Larissa Murr, Melanie Pavlovic, Annika Sprenger, Mareike Wenning, Thomas Alter, Ute Messelhäußer

**Affiliations:** 1 Bavarian Health and Food Safety Authority, Department Erlangen and Oberschleißheim, Veterinärstraße 2, 85764 Oberschleißheim, Germany; 2 Institute of Food Safety and Food Hygiene, Freie Universitaet Berlin, 14195 Berlin, Germany

**Keywords:** *Listeria monocytogenes*, whole-genome sequencing, antimicrobial resistance, virulence factors, soft fruit

## Abstract

Reported cases of listeriosis from food of non-animal origin (FNAO) are increasing. In order to assess the risk of exposure to *Listeria monocytogenes* from FNAO, the genetic characterization of the pathogen in FNAO products and in primary production and processing plants needs to be investigated. For this, 123 samples of fresh and frozen soft fruit and 407 samples of 39 plants in Bavaria, Germany that produce and process FNAO were investigated for *Listeria* contamination. As a result, 64 *Listeria* spp. isolates were detected using ISO 11290-1:2017. Environmental swabs and water and food samples were investigated. *L. seeligeri* (36/64, 56.25%) was the most frequently identified species, followed by *L. monocytogenes* (8/64, 12.50%), *L. innocua* (8/64, 12.50%), *L. ivanovii* (6/64, 9.38%), *L. newyorkensis* (5/64, 7.81%), and *L. grayi* (1/64, 1.56%). Those isolates were subsequently sequenced by whole-genome sequencing and subjected to pangenome analysis to retrieve data on the genotype, serotype, antimicrobial resistance (AMR), and virulence markers. Eight out of sixty-four *Listeria* spp. isolates were identified as *L. monocytogenes*. The serogroup analysis detected that 62.5% of the *L. monocytogenes* isolates belonged to serogroup IIa (1/2a and 3a) and 37.5% to serogroup IVb (4b, 4d, and 4e). Furthermore, the MLST (multilocus sequence typing) analysis of the eight detected *L. monocytogenes* isolates identified seven different sequence types (STs) and clonal complexes (CCs), i.e., ST1/CC1, ST2/CC2, ST6/CC6, ST7/CC7, ST21/CC21, ST504/CC475, and ST1413/CC739. The core genome MLST analysis also showed high allelic differences and suggests plant-specific isolates. Regarding the AMR, we detected phenotypic resistance against benzylpenicillin, fosfomycin, and moxifloxacin in all eight *L. monocytogenes* isolates. Moreover, virulence factors, such as *prfA*, *hly*, *plcA*, *plcB*, *hpt*, *actA*, *inlA*, *inlB,* and *mpl*, were identified in pathogenic and nonpathogenic *Listeria* species. The significance of *L. monocytogenes* in FNAO is growing and should receive increasing levels of attention.

## 1. Introduction

The public health sector has had to increasingly focus on foodborne illnesses over the last decade [1]. Microbiologically contaminated food, in particular, plays a decisive role. Next to *Escherichia coli*, *Salmonella* spp., and *Campylobacter*, *Listeria monocytogenes* has caused severe foodborne diseases [2]. *L. monocytogenes* records one of the highest mortality rates in humans (20–30%) with regard to invasive listeriosis in vulnerable population groups [3]. Food of animal origin (FAO), such as meat, milk, and fish products, is commonly known as a vehicle for *L. monocytogenes* [4], but the listeriosis cases associated with food of non-animal origin (FNAO) have been increasing in recent years [5,6,7,8,9,10,11]. Diced celery, packaged salads, stone fruit, sprouts, soy products, whole apples, cantaloupes, frozen vegetables, and, in particular, frozen corn were found to have been contaminated with *L. monocytogenes* inducing listeriosis [5,6,7,8,9,10,11,12]. One of the largest and first widespread plant-based listeriosis outbreaks in the United States was caused by cantaloupes in 2011. During this outbreak, 147 cases including 143 hospitalizations and 33 deaths were reported in 28 different states [5,13]. The melons were most likely contaminated by the company’s environment [13]. This underlines the risk associated with FNAO, especially ready-to-eat (RTE) products that do not undergo further heating or processing steps. Furthermore, frozen corn and probably other frozen vegetables (vegetables mixes, green beans, and spinach) were responsible for 47 human listeriosis cases and a 19% fatality rate in the European Union up to June 2018 [8]. As with the *L. monocytogenes* isolates found in cantaloupes, *L. monocytogenes* isolates from frozen corn and vegetables have also been found in the facilities’ environments [8,13]. This fact accentuates the need for appropriate disinfection and cleaning interventions in FNAO-producing companies. Furthermore, the annual European Union One Health Zoonoses Report showed *L. monocytogenes* in fruits and vegetables as a more pervasive issue [14,15]. From 2017 to 2021, the sampling units and, therefore, the prevalence rate increased as well [15]. Therefore, the topic of FNAO and *L. monocytogenes* will continue to gain importance in the sector.

In order to assess the risk of exposure to *L. monocytogenes* from FNAO, the occurrence and further characteristics, such as genotype and serotype detection, antimicrobial resistances (AMR), and virulence markers of the pathogen in FNAO products and primary production and processing plants, needs to be investigated. Up to now, only a few studies examined the presence and further characteristics of *L. monocytogenes* in different areas of FNAO production and processing companies [16,17,18]. In addition, the more isolates from agricultural environments, FNAO fresh produce, and RTE food and processing environments that are collected and characterized, the greater the understanding of foodborne-associated outbreaks related to *L. monocytogenes* [19].

The linkage between human isolates and food as the transmission vehicle was revolutionized through the development of next-generation sequencing technologies [20]. Over the last few years, whole-genome sequencing (WGS) has become more important in characterizing and connecting different isolates of *L. monocytogenes* in a wide range of food production [19,21,22]. To obtain more knowledge on the relationship of *Listeria* spp., especially *L. monocytogenes*, isolates, and FNAO, 64 *Listeria* spp. isolates were detected from FNAO products and the companies that produce and process these products. The isolates were sequenced by WGS and subjected to pangenome analysis.

## 2. Materials and Methods

The sampling scheme, number of samples, and *Listeria* spp. detection methods are already described by Wartha et al. [23]. In addition to the 407 samples from 39 primary production and processing companies already described [23], 123 samples of fresh fruit and frozen berries from supermarkets were collected and analyzed. The study of Wartha et al. [23] was furthermore extended with WGS results of 64 detected *Listeria* spp. and *L. monocytogenes* isolates from FNAO products from supermarkets and FNAO-producing and -processing plants.

### 2.1. Origin of Isolates from Supermarkets

In this study, 123 samples of fresh soft fruit and frozen berries were collected randomly from supermarkets across the south of Bavaria, Germany. The sampling period was between January 2021 and April 2021 and the samples came from different countries (including Canada, Chile, Greece, Mexico, Morocco, Peru, Poland, Portugal, Serbia, Spain, The Netherlands, and the USA). Blueberries, raspberries, strawberries, and blackberries were included in the 63 fresh soft fruit samples. Furthermore, 60 samples of frozen raspberries, blueberries, blackberries, strawberries, currants, cherries, and cranberries (partially mixed together) were included among the frozen berries category. The frozen samples were prepared in three different ways before examination; the first group was thawed for 3 h at room temperature, the second group was defrosted for 16 h in the refrigerator, and the third group was tested frozen. The samples were analyzed using methods described in ISO 11290-1:2017 [24].

### 2.2. Origin of Isolates from FNAO-Producing and -Processing Plants

During the on-the-spot verification visits, 407 samples were taken from 39 FNAO primary producing and processing companies in Bavaria, Germany from July 2020 until June 2021. Thirty plants were categorized as primary production (i.e., farm and primary production level) and nine companies as processing companies (i.e., processing level). The 407 samples included 229 swab samples (food contact material and environment), 59 food samples (soft fruit, vegetables, RTE vegetables, and other food samples), and 119 irrigation and processing water samples [23]. For the detection of *Listeria* spp. and *L. monocytogenes*, ISO 11290–1:2017 [24] was used as described [23]. 

### 2.3. WGS

For sequencing, the DNA was extracted using the Invitrogen PureLink Genomic DNA Mini Kit (Thermo Fisher Scientific, Waltham, MA, USA), which is suitable for Gram-positive bacteria, with slight modifications. Particularly, blood agar plates (Oxoid Deutschland GmbH, Wesel, Germany) were incubated at 37 °C for 24 ± 2 h. One third of the bacterial colony overgrown on the blood agar plate was diluted in 200 µL 1× phosphate-buffered saline (PBS; Biochrom AG, Berlin, Germany). After the first centrifugation step (20,000× *g* for 5 min), the supernatant was removed. A second centrifugation step (20,000× *g* again briefly) was performed to remove all of the supernatant. The pellet was used for further DNA extraction steps. Furthermore, instead of the PureLink Genomic Elution Buffer, the EDTA free elution buffer from the QIAquick PCR Purification Kit (Qiagen GmbH, Hilden, Germany) was used. After purification, the DNA purity was measured with the Nanodrop 1000 Spectrophotometer (Thermo Fisher Scientific). The DNA concentration was determined either with a Quantus Fluorometer (Promega Corporation, Madison, WI, USA) with the QuantiFluor ONE dsDNA System kit (Promega Corporation) or a Qubit 3.0 Fluorometer (Thermo Fisher Scientific) with the Qubit dsDNA BR Assay kit (Thermo Fisher Scientific). Lambda DNA (Promega Corporation) was used as the standard. Before preparing the dual-indexed paired-end libraries, all samples were diluted to 24 ng [25]. In preparing the libraries, Illumina DNA Prep (Illumina Inc., San Diego, CA, USA) was used with a few modifications. Instead of Sample Purification Beads (SPB; Illumina Inc.), Agencourt AMPure XP Beads (Beckman Coulter GmbH, Krefeld, Germany) were used during the final clean-up step. After the concentration measurements with the Quantus Fluorometer and the associated QuantiFluor ONE dsDNA System kit (Promega Corporation), the libraries’ quality and fragment size distribution were checked on a 5200 Advanced Analytical Fragment Analyzer (Agilent Technologies Inc., Santa Clara, CA, USA) using the HS NGS High Sensitivity 474 kit (Agilent Technologies Inc.) as instructed by the manufacturer. The Fragment Analyzer data were edited using the ProSize Data analysis software, version 4.0.2.7 (Agilent Technologies Inc.). Library normalization was performed using the Biomek i7 Automated Workstation (Beckman Coulter GmbH). After pooling the libraries, the denaturation and dilution steps were performed following the NextSeq System Denature and Dilute Libraries Guide Protocol A (Illumina Inc.), as the manual instructions required. The denaturated library solution was diluted to 1.3 pM. The sequencing was performed on a NextSeq 550 using a Mid Output Reagent Cartridge v2 for 300 cycles (Illumina Inc.). The read length was 2 × 149 bp, and PhiX (1%, Illumina Inc.) was used as the sequencing control.

The raw sequencing data were uploaded to the Sequence Read Archive (SRA) of the National Center for Biotechnology Information (NCBI) database (https://www.ncbi.nlm.nih.gov/sra accessed on 28 February 2023) under the project number PRJNA935401.

### 2.4. Data Analysis

The AQUAMIS pipeline v1.2.0 (Bundesinstitut für Risikobewertung, BfR, Berlin, Germany) was used for the trimming of the sequencing reads, read assembly, and overall quality analysis [26]. The generated assemblies were annotated using Prokka v1.14.6 [27]. Subsequently, the pangenome was calculated with PIRATE v1.0.3 [28]. The dendrogram was generated using PIRATE v.1.0.3 with the default parameters based on the core-genome alignments [29]. Metadata such as the holdings of origin were added with iTOL [30]. Moreover, the assemblies were screened for the presence of virulence and AMR genes with abricate v1.0.1 [31], utilizing the data from the Comprehensive Antibiotic Resistance Database (CARD) [32] and the virulence factor database (VFDB) [33]. For the serogroup detection, a serogroup scheme (v1.0) of five loci [34] was used, and for the MLST (multilocus sequence typing) a seven loci MLST scheme (v1.0) [35] was used, both integrated in the Ridom SeqSphere+ Software (v 8.3.1) [36]. The used scheme for serogrouping allowed the classification into the following serogroups: IIa (1/2a and 3a), IIb (1/2b), IIc (1/2c and 3c), and IVb (4b, 4d and 4e) [37]. The core genome MLST (cgMLST) was performed for the *L. monoyctogenes* isolates using the 1701 loci scheme [38] (v 2.1) integrated in the Ridom SeqSphere+ Software (v 8.3.1) [37]. The cgMLST minimum spanning tree (MST) was generated based on the core genome targets integrated in the Ridom SeqSphere+ Software (v 8.3.1) [36].

### 2.5. Phenotypic AMR Testing

For the phenotypic characterization, the isolates were incubated at 37 °C for 24 ± 2 h on blood agar (Oxoid Deutschland GmbH). For the AMR testing, the BD Phoenix System (Becton Dickinson, Franklin Lakes, NJ, USA) with the PMIC/ID 88 panel was used, according to the manufacturer’s guidance for Gram-positive bacteria. The phenotypic resistance of 22 *Listeria* spp. isolates against benzylpenicillin, erythromycin, trimethoprim-sulfamethoxazole, gentamicin, tetracycline, fosfomycin, ciprofloxacin, and moxifloxacin was tested according to the European Committee on Antimicrobial Susceptibility Testing (EUCAST). The EUCAST breakpoints for *L. monocytogenes* were used for erythromycin and trimethoprim–sulfamethoxazole [39], whereas, for the other tested antibiotics (i.e., benzylpenicillin, gentamicin, tetracycline, fosfomycin, ciprofloxacin, and moxifloxacin), the EUCAST *Staphylococcus* spp. breakpoints were applied as a substitute, as described in previous studies [40,41].

## 3. Results

In summary, this study examined 64 Listeria spp. isolates in total (63 isolates from 39 FNAO-producing and -processing plants and one isolate from 123 FNAO samples from supermarkets). *L. seeligeri* (36/64, 56.25%) was the most frequently identified species, followed by *L. monocytogenes* (8/64, 12.50%), *L. innocua* (8/64, 12.50%), *L. ivanovii* (6/64, 9.38%), *L. newyorkensis* (5/64, 7.81%), and *L. grayi* (1/64, 1.56%).

### 3.1. Occurrence of Listeria spp. in FNAO from Supermarkets

A total of 123 samples from fresh and frozen soft fruit taken from randomly chosen supermarkets in the south of Bavaria were investigated. *L. grayi* (1/123, 0.81%) was detected in a frozen blackberry package. All of the other 122 samples showed no presence of *Listeria* spp., including *L. monocytogenes*. Accompanying bacterial and fungal flora, such as moulds, yeasts, enterococci, and bacilli, were noticed. Furthermore, the analytical approach of preparing the frozen soft fruit samples in three different ways before examination (frozen, refrigerator, or room temperature) showed no differences in the results and revealed no impact on detecting *Listeria* spp. or *L. monocytogenes* in this study. 

### 3.2. Occurrence of Listeria spp. in FNAO-Producing and Processing Plants

Overall, 407 samples from 39 FNAO primary production and processing facilities were investigated, as described previously [23]. Of the companies visited, 48.72% (19/39) of the samples tested positive for *Listeria* spp. In 42.11% (8/19) of the samples from these facilities, only one *Listeria* spp. was detected. In comparison, more than one *Listeria* spp. was identified in 57.89% (11/19) of the samples from FNAO plants. Eight *L. monocytogenes* isolates were identified in six environmental swabs and two processing water samples. No food sample tested positive for *L. monocytogenes*.

### 3.3. Genetic Relation of 64 Listeria Isolates Sequenced by WGS

Overall, 64 *Listeria* isolates were sequenced by WGS and subjected to pangenome analysis. The coverage depth of the sequencing ranged from a minimum of 30.6-fold to a maximum coverage of 107-fold. In the 64 assemblies, a total of 8475 gene families were found, with 1588 of these presenting in at least 95% of the assemblies.

The different isolates were grouped according to their species. However, the isolates within the same species and from the same company differed from each other. For example, the *L. seeligeri* isolates (SWB9, SWA9, and SWC4) were isolated from processing water for a salad washing system, and SWC4 differed from SWB9 and SWA9. Moreover, the *L. ivanovii* isolates (SWC3 and SWC7) from a drain differed from each other, whereas the isolate SWD3 from a delight mixed salad from company A was more similar to the isolate SWC7. The dendrogram, which depicts the genetic relationships and phylogeny among the 64 *Listeria* spp. isolates, is provided in the Supplemental Materials (Figure S1).

### 3.4. Serogroup Determination of L. monocytogenes

Eight isolates of *L. monocytogenes* were detected in six various plants, with four at the processing level and four at the production level. Table 1 shows that the *L. monocytogenes* isolates belonged to lineages I and II, with 62.5% (5/8) of the isolates belonging to serogroup IIa (comprising serovars 1/2a and 3a) and 37.5% (3/8) to serogroup IVb (serovars 4b, 4d, and 4e).

### 3.5. MLST Analysis of L. monocytogenes

Seven different sequence types (STs) and clonal complexes (CCs) were identified, namely, ST1/CC1, ST2/CC2, ST6/CC6, ST7/CC7, ST21/CC21, ST504/CC457, and ST1413/CC739 (Table 1). The isolates SWD4 and SWH4 (a single processing water sample for a salad washing system) showed the same ST and CC combination.

### 3.6. cgMLST Analysis of L. monocytogenes

**Figure 1:**
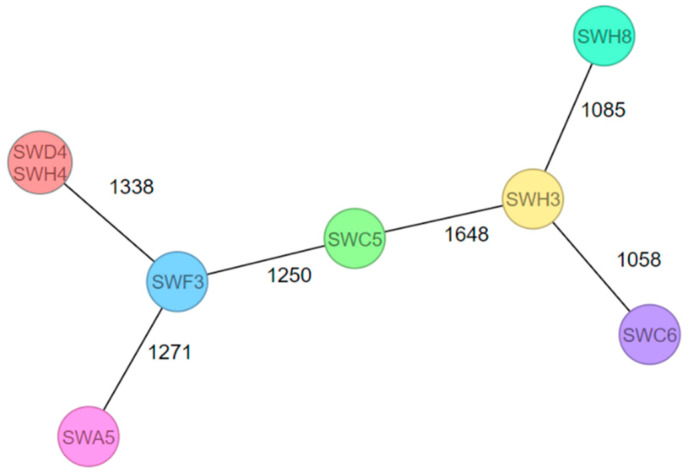
cgMLST MST of eight *L. monocytogenes* isolates from drains (SWH8, SWC6, SWH3, SWC5, SWF3, and SWA5) and from processing water for a salad-washing system (SWD4 and SWH4). The allele distances are indicated. Identical isolates are grouped within a common circle.

Figure 1 shows the cgMLST MST for the eight detected *L. monocytogenes* isolates. With the exception of the two isolates SWD4 and SWH4, the *L. monocytogenes* isolates differed by 1058 to 1648 alleles. Moreover, seven different complex types (CTs) were identified (Table 1). The isolates SWH8 (CT16889), SWD4 (CT16886), and SWH4 (CT16886) originated from the same primary production plant, but isolate SWH8 showed a different CT compared to the isolates SWD4 and SWH4. However, SWD4 and SWH4 were isolated from the same sample. 

### 3.7. Prevalence of Genetic and Phenotypic AMRs in Listeria spp.

Sixty-four created assemblies were used for further investigations, namely, AMR and virulence gene analysis. In 17 isolates, five AMR genes (antibiotic class) were detected, namely, *lin* (lincomycin), *norB* (fluoroquinolone), *fosX* (fosfomycin), *tetM* (tetracycline), and *ANT(6)-la* (aminoglycosides). Additionally, *mprF*, a gene encoding an integral membrane protein with resistance to cationic peptides that disrupt the cell membrane, including defensins, was detected [33]. The presence of the AMR genes is shown in Table 2. AMR genes were detected in eight *L. monocytogenes* isolates, eight *L. innocua,* and one *L. seeligeri* isolate. The phenotypic characterization was performed with 22 *Listeria* spp. isolates: 17 isolates harboring AMR genes (Table 2) and 5 *L. newyorkensis* isolates to obtain more information on the antimicrobial susceptibility of this species (Table 3). The associated minimum inhibitory concentration (MIC) results are shown in the Supplemental Materials (Table S1). All *L. newyorkensis* isolates were from swabs (drains) and water samples (a single processing water sample for lettuce and an irrigation water sample for vegetables). Furthermore, the genetic AMR results of *L. monocytogenes* differed from the phenotypical AMR results. More phenotypical AMR findings were detected in the *L. innocua* and *L. seeligeri* isolates than genetic resistances identified (Table 2).

### 3.8. Prevalence of Virulence Genes in Listeria spp.

Table 4 demonstrates the presence of different virulence genes. All eight *L. monocytogenes* isolates carried *prfA*, which regulates the production of virulence factors [42]. Consequently, the eight sequenced *L. monocytogenes* isolates carried the virulence genes, which are regulated by *prfA*: *hly*, *plcA*, *plcB*, *hpt*, *actA*, *inlA,* and *inlB* (Table 4) [43]. The *llsX* gene belonging to LIPI (*Listeria* pathogenicity island)-3 was detected in two *L. monocytogenes* isolates belonging to lineage I (SWC6 and SWH3). 

Furthermore, the *prfA*, *hly*, *plcA*, *plcB,* and *mpl* genes were detected in 33 *L. seeligeri* isolates (every isolate except SWE1, SWH7, and SWH10). The *hpt* gene was detected in every *L. seeligeri* isolate, as well as in every *L. ivanovii* isolate. Four isolates of *L. ivanovii* (SWA3, SWC7, SWD3, and SWG2) carried the genes *prfA*, *hly*, *inlA,* and *inlB*. *L. innocua*, *L. newyorkensis,* and *L. grayi* did not show any of the selected virulence factor genes.

## 4. Discussion

### 4.1. Distribution and Genetic Relation of Listeria spp. in FNAO from Supermarkets and FNAO-Producing and Processing Plants

The study showed that *Listeria* spp. were spread among 48.72% (19/39) of the FNAO facilities in Bavaria. The distribution and genetic heterogeneity of the isolates within the species and the companies (Figure S1) suggests different contamination pathways of *Listeria* spp. and the introduction from the environment or irrigation and processing water. As the genus *Listeria* is ubiquitous in the environment [48], incoming raw goods, irrigation and processing water, coworkers, or working utensils are potential sources of contamination [48,49]. In particular, incoming raw material is described as a key determinant [49]. Less similar isolates are less likely to share a recent common ancestor [50]. The different values of *L. monocytogenes* CTs from the same company (Table 1) support the hypothesis of *L. monocytogenes* introduction from the environment. Nevertheless, once *L. monocytogenes* and other *Listeria* spp. have entered the processing plant, the likelihood of persisting increases [51,52]. In addition to good hygienic practice, periodical environmental sampling helps to obtain an overview of the prevalence and persistence of *Listeria* spp. and *L. monocytogenes* within a plant [49].

### 4.2. Serogroup and MLST Analysis of L. monocytogenes

From 2007 to 2015, the EFSA reported an 87% prevalence of serogroup IIa and IVb in the EU [26]. This is confirmed by our results, as the *L. monocytogenes* isolates in this study were classified into serogroups IIa (1/2a and 3a) (62.5%, 5/8) and IVb (4b, 4d, and 4e) (37.5%, 3/8). They were sampled from the environments of the FNAO companies (Table 1) [53]. An isolate detected in the condensate ponding of a cooling unit (SWC6) belonged to serogroup IVb and ST6 (Table 1). *L. monocytogenes* of serogroup IVb and ST6 was already responsible for an outbreak that impacted five different countries. As of June 2018, the case fatality rate was 19% with 47 reported cases, and the causative food was frozen corn and frozen vegetables [8]. 

Furthermore, the *L. monocytogenes* isolates with ST1/CC1, ST2/CC2, ST7/CC7, and ST21/CC21 identified in our study were detected in various drains at FNAO primary production and processing plants [54,55,56,57]. ST1 and ST2 were reported as hypervirulent by Mafuna et al. and well-adapted to persist in food processing environments in South Africa [54]. ST7/CC7 was reported as one of the most relevant agents of cattle abortions in Latvia [55]. Furthermore, ST21 was associated with *L. monocytogenes* isolated from vegetables by Cabal et al. [56]. ST21/CC21 was identified exclusively in 2011 and 2014 from environmental swab samples in a small meat processing facility in Montenegro [57]. This demonstrates that the detected isolates in our study were already described and showed diverse distribution.

### 4.3. cgMLST Analysis of L. monocytogenes

The created MST (Figure 1) shows high allelic differences between the eight detected *L. monocytogenes* isolates from the environment of FNAO-producing and -processing companies. These results suggest plant-specific *Listeria* isolates with sporadic introduction. However, due to the low number of positive samples and missing data of periodical sampling, it is difficult to generalize and extrapolate the statement. In 2011, contaminated cantaloupes were responsible for a listeriosis outbreak in 28 different states in the US. One single company was the starting point for this listeriosis outbreak [58]. Furthermore, investigations into the context of a listeriosis outbreak caused by ice cream products in the United States revealed operation-related food product contamination as well. The human *L. monocytogenes* isolates matched with the food product (ice cream) isolated from a production line in a specific company [59]. A comparison of ice cream products from another producing facility operated by the same brand showed no accordance with the listeriosis outbreak [60]. In comparison, other studies detected related *L. monocytogenes* isolates from meat (e.g., poultry or pork) in geographically independent producing plants [61,62], which suggests food matrix-dependent and persisting *L. monocytogenes* isolates in the meat sector. The finding of no genetically related *L. monocytogenes* isolates in the environment of the FNAO-producing and -processing companies suggested that there was no connection among the plants. Isolates that were linked to the producing and processing company and geographically dependent isolates in the FNAO sector were suggested. No transfer of *L. monocytogenes* among Bavarian FNAO-producing and -processing companies’ environment to food products has taken place.

### 4.4. Genetic and Phenotypic AMRs of Listeria spp.

AMR has already become a global issue [63]. In 1988, AMR against erythromycin, tetracycline, chloramphenicol, and streptomycin was already reported in *L. monocytogenes* [64]. The detection of the genes *fosX*, *lin*, *norB,* and *mprF* in *L. monocytogenes* isolates was identical with the results of Parra-Flores et al. from RTE food in Chile [65]. The lincomycin resistance, as indicated by the presence of the *lin* gene, was not possible to confirm phenotypically, because the BD-Phoenix-System PMIC/ID 88 panel for *Staphylococcus* spp. did not include the lincomycin antibiotic. Furthermore, neither the EUCAST *L. monocytogenes* nor the *Staphylococcus* spp. breakpoints show MIC breakpoints for lincomycin [39]. In our study, every *L. monocytogenes* isolate showed phenotypic resistance to benzylpenicillin (Table 3). Tîrziu et al. detected *L. monocytogenes* isolates out of FAO, all of which showed benzylpenicillin resistance [66]. Furthermore, benzylpenicillin resistance in isolates from RTE vegetables was documented [67]. Penicillin is used as an antibiotic against listeriosis [41], and a 100% AMR detection rate in our study is a cause of concern. However, our results showed a phenotypically detected benzylpenicillin resistance without an underlying antibiotic gene. The lack of suitable breakpoints may explain the discrepancies between the phenotypic and genotypic antimicrobial susceptibility data [68].

Genotypic and phenotypic resistances to fluoroquinolones (here, ciprofloxacin and moxifloxacin were tested) were shown (Table 2), and ciprofloxacin resistance was reported in other studies [41,66,69]. We detected the *norB* gene that confers resistance to fluoroquinolones with an antibiotic efflux mechanism [33]. Other studies described *lde* as an underlying antibiotic gene for AMR to fluoroquinolones in *L. monocytogenes* [70,71]. Developing acquired AMR is rarely described in *L. monocytogenes* isolates, but Morvan et al. showed AMR of *L. monocytogenes* to fluoroquinolones, which suggests acquired AMR even if remaining low [71]. Acquired AMR to tetracycline was shown as well [71].

Moreover, the gene *fosX* and resistance to fosfomycin was detected (Table 2). Scortti et al. described that, despite *fosX* gene expression, *L. monocytogenes* isolates were susceptible to the antibiotic fosfomycin due to epistasis, but only during infection [72]. On the other hand, a natural in vitro resistance of *L. monocytogenes* to fosfomycin has been reported as well [41]. 

*L. innocua*, *L. seeligeri,* and *L. newyorkensis* showed AMR as well (Table 3), which suggests *Listeria* spp. as a habitat of AMR genes [73]. Different studies detected *L. innocua* as the species that is less susceptible to antibiotics compared to other *Listeria* spp. and reduced sensitivity against benzylpenicillin, tetracycline, fosfomycin, and ciprofloxacin, as our results confirmed [73,74,75]. Potential resistance gene transfer in *Listeria* spp. was discussed and increases the risk of emerging AMR in *L. monocytogenes* [73,75]. 

In general, the comparability of the AMR data is limited due to the different methods used [69] and the small number of isolates tested in our study. Furthermore, this study showed that the genotypic AMR results differed from the phenotypic AMR results. Under the heading of intrinsic antibiotic resistome, it is possible that phenotypical characteristics were influenced by bacterial metabolism, i.e., inactivation of genes, which changes the bacterial efficacy to antibiotics [76]. On the other hand, revisions of the threshold values were recommended to avoid misclassifying susceptibilities [68], as this may explain the differences between the genotypic and phenotypic susceptibility data. Gygli et al. described that clinical concentrations of antimicrobial susceptibility testing of *Mycobacterium tuberculosis* were defined too high and, thus, misclassifying and discrepancies between the genotypic and phenotypic data occurred [68]. The linkage of genotypic and phenotypic and a continuous surveillance of AMR could improve the understanding of AMR expressions [68,71].

Until now, *L. newyorkensis* was isolated from a milk processing company in the state of New York and from river water in Japan [77,78]. In our study, *L. newyorkensis* was isolated from swabs (drains) and water samples (a single processing water sample for lettuce and an irrigation water sample for vegetables). To the best of our knowledge, there is no literature concerning AMR in *L. newyorkensis* so far. Our results showed phenotypic resistances against benzylpenicillin, erythromycin, and fosfomycin according to the EUCAST *Staphylococcus* spp. breakpoints. However, no underlying genetic resistances were detected in the five *L. newyorkensis* isolates.

### 4.5. Prevalence of Virulence Genes in Listeria spp.

The presence of virulence genes is presented in Table 4. *L. monocytogenes* isolates exhibited the *prfA* gene, which is responsible for further virulence gene expression [79]. However, premature stop codon mutations in *inlA* in lineage II *L. monocytogenes* strains [53,80] were identified and suggest less invasiveness in 62.5% (5/8) of our detected *L. monocytogenes* isolates. Quereda et al. described that LIPI-3 is present in 50% of *L. monocytogenes* lineage I isolates, and LIPI-4 was present in CC4 isolates [81]. The *llsX* gene, belonging to LIPI-3, was detected in two *L. monocytogenes* isolates. A strong relationship between *llsX* and the invasiveness of *L. monocytogenes* is discussed [82]. LIPI-4 has been detected in CC4 isolates [81], which were not identified in this study. However, the *L. monocytogenes* singleton ST382 was responsible for multistate outbreaks linked to FNAO (caramel apples, stone fruit, and packaged leafy green salad) and carried LIPI-4 [83]. Furthermore, Disson et al. described LIPI-4 in *L monocytogenes* CC87, as well as in *L. innocua* isolates [84].

In addition to *L. monocytogenes*, *L. ivanovii* was reported as a pathogenic species of *Listeria* [51,85,86]. Gouin et al. described that *L. ivanovii* and *L. seeligeri* carried genes of the virulence gene cluster of *L. monocytogenes* (i.e., *prfA*, *plcA*, *hly*, *mpl*, *actA,* and *plcB*) [86]. Our results showed that every *L. ivanovii* isolate carried the *hpt* gene, and four out of six isolated *L. ivanovii* isolates carried the *prfA*, *hly*, *inlA*, and *inlB* genes (Table 4). The *inlA* gene encoding for the *inlA* protein is necessary for entering the host cell [87] and was present in four *L. ivanovii* isolates. However, experimental assays showed that the *L. ivanovii* isolates that possessed the protein *inlA* showed less invasion in human cells compared to *L. monocytogenes*, and human listeriosis cases caused by *L. ivanovii* were rare as well [85]. Additionally, the absence of *plcA*, *mpl*, *actA,* and *plcB* suggested a missing virulence potential [88].

Furthermore, 56.25% (36/64) of the detected *Listeria* spp. were assigned to *L. seeligeri*. The gene *hpt* was present in every *L. seeligeri* isolate (Table 4). Our results showed that 91.67% of the detected *L. seeligeri* isolates were verified, with five virulence genes, namely, *prfA*, *hly*, *pclA*, *plcB,* and *mpl*. However, the *actA*, *inlA*, and *inlB* genes were not present. On the basis of the WGS analysis and because of the temporal and spatial dependency of virulence gene regulation, it is unlikely that the *L. seeligeri* isolates in our study had virulent potential [89]. Nevertheless, despite experimental studies [90,91] concluding that the species *L. seeligeri* is nonpathogenic, a single human meningitis case caused by *L. seeligeri* was reported in Switzerland [92].

## 5. Conclusions

In conclusion, this study showed that *Listeria* spp., including the pathogenic *L. monocytogenes*, were present in FNAO plants in Bavaria, Germany, but no food sample tested positive for *L. monocytogenes*. The genetic differences suggested external introduction, diverse ancestors, and plant-specific distribution of *L. monocytogenes* in FNAO-producing and -processing plants. One identified *L. monocytogenes* isolate in our study belonged to serogroup IVb and ST6. *L. monocytogenes* serogroup IVb and ST6 isolated from frozen corn and other frozen vegetables was already responsible for a multi-country outbreak. Furthermore, isolates with virulence markers and antibiotic resistances were identified, which should not be underestimated. However, further periodical sampling could provide more insights into persisting isolates.

## Figures and Tables

**Table 1:** Characterization of the *L. monocytogenes* isolates from FNAO plants.

Isolate	Plant (PP or PC)	Sample	Serogroup and Serotype (In Silico)	Sequence Type (ST) (MLST)	Clonal Complex (CC) (MLST)	Complex Type (CT) (cgMLST)
SWH3	K (PC)	Drain—delivery raw goods (S)	IVb (4b, 4d, and 4e)	ST1	CC1	16888
SWH8	I (PP)	Drain—cooling area lettuce (S)	IVb (4b, 4d, and 4e)	ST2	CC2	16889
SWC6	M (PP)	Condensate ponding of a cooling unit (S)	IVb (4b, 4d, and 4e)	ST6	CC6	7504
SWA5	N (PC)	Drain—frozen food packaging (S)	IIa (1/2a and 3a)	ST7	CC7	16884
SWF3	G (PC)	Drain—packaging area lettuce (S)	IIa (1/2a and 3a)	ST21	CC21	16887
SWD4	I (PP)	Processing water for a salad-washing system (PW)	IIa (1/2a and 3a)	ST504	CC475	16886
SWH4	I (PP)	Processing water for a salad-washing system (PW)	IIa (1/2a and 3a)	ST504	CC475	16886
SWC5	E (PC)	Drain—prewashing area (S)	IIa (1/2a and 3a)	ST1413	CC739	16885

*L. monocytogenes* isolates were detected in six different plants (N, E, M, G, K, and I). The sample types were swabs (S) and processing water (PW). Four *L. monocytogenes* isolates were found at processing companies (PCs, *n* = 9) and four *L. monocytogenes* isolates at primary production plants (PPs, *n* = 30). The isolates SWD4 and SWH4 were from the same sample.

**Table 2:** AMR genes and phenotypically detected resistances of 17 *Listeria* isolates.

No.	Isolate	*Listeria* spp.	Sample	Resistance Gene(s)	Resistance	Phenotypical Resistance
1	SWA5	*L. monocytogenes*	Swab	*fosX, mprF, lin, norB*	Fosfomycin, cationic peptide, lincomycin, fluoroquinolones	PEN, FOS, MOX
2	SWC5	*L. monocytogenes*	Swab	*fosX, mprF, lin, norB*	Fosfomycin, cationic peptide, lincomycin, fluoroquinolones	PEN, FOS, CIP, MOX
3	SWC6	*L. monocytogenes*	Swab	*fosX, mprF, lin, norB*	Fosfomycin, cationic peptide, lincomycin, fluoroquinolones	PEN, GEN, FOS, MOX
4	SWH8	*L. monocytogenes*	Swab	*fosX, mprF, lin, norB*	Fosfomycin, cationic peptide, lincomycin, fluoroquinolones	PEN, FOS, CIP, MOX
5	SWH3	*L. monocytogenes*	Swab	*fosX, mprF, lin, norB*	Fosfomycin, cationic peptide, lincomycin, fluoroquinolones	PEN, FOS, CIP, MOX
6	SWF3	*L. monocytogenes*	Swab	*fosX, mprF, lin, norB*	Fosfomycin, cationic peptide, lincomycin, fluoroquinolones	PEN, FOS, CIP, MOX
7	SWD4	*L. monocytogenes*	Water	*fosX, mprF, lin, norB*	Fosfomycin, cationic peptide, lincomycin, fluoroquinolones	PEN, FOS, MOX
8	SWH4	*L. monocytogenes*	Water	*fosX, mprF, lin, norB*	Fosfomycin, cationic peptide, lincomycin, fluoroquinolones	PEN, FOS, CIP, MOX
9	SWA11	*L. innocua*	Swab	*norB*	Fluoroquinolones	PEN, FOS, CIP, MOX
10	SWB4	*L. innocua*	Swab	*norB*	Fluoroquinolones	PEN, FOS, CIP, MOX
11	SWB11	*L. innocua*	Swab	*norB*	Fluoroquinolones	PEN, Trim-sulfa, FOS, CIP, MOX
12	SWA1	*L. innocua*	Swab	*norB*	Fluoroquinolones	PEN, FOS, CIP, MOX
13	SWF1	*L. innocua*	Swab	*norB*	Fluoroquinolones	PEN, FOS, CIP, MOX
14	SWF5	*L. innocua*	Food	*norB*	Fluoroquinolones	PEN, FOS, CIP, MOX
15	SWG7	*L. innocua*	Food	*norB*	Fluoroquinolones	PEN, FOS, CIP, MOX
16	SWH6	*L. innocua*	Swab	*norB, tetM, ANT(6)-la*	Fluoroquinolones, tetracycline, aminoglycoside nucleotidyltransferase	PEN, Trim-sulfa, GEN, TET, FOS, CIP, MOX
17	SWG3	*L. seeligeri*	Swab	*norB*	Fluoroquinolones	PEN, FOS, CIP, MOX

*Lin* = *L monocytogenes* EGD-e line gene for the lincomycin resistance ABC-F-type ribosomal protection protein of complete CDS; *norB* = a multidrug efflux pump in *Staphylococcus aureus* that confers resistance to fluoroquinolones and other structurally unrelated antibiotics such as tetracycline; *fosX* = an enzyme used to confer resistance to fosfomycin, which is dependent on the cofactor manganese (II) and uses water to generate a vicinal diol; *mprF* = an integral membrane protein that modifies the negatively charged phosphatidylglycerol on the membrane surface. This confers resistance to cationic peptides that disrupt the cell membrane including defensins; *tetM* = a ribosomal protection protein that confers tetracycline resistance. It is found on transposable DNA elements, and its horizontal transfer between bacterial species has been documented; *ANT(6)-Ia* = an aminoglycoside nucleotidyltransferase gene encoded by plasmids and chromosomes in *Staphylococcus epidermidis, E. faecium, Streptococcus suis, S. aureus, E. faecalis,* and *Streptococcus mitis*. All gene descriptions were defined by CARD [33]; PEN = benzylpenicillin; GEN = gentamicin; FOS = fosfomycin; CIP = ciprofloxacin; MOX = moxifloxacin; Trim-sulfa = trimethoprim-sulfamethoxazole; TET = tetracycline. The five *L. newyorkensis* isolates are not included in this table.

**Table 3:** Phenotypical characterization of 22 *Listeria* isolates.

Antibiotics	MIC Breakpoints (mg/L)		Number of Resistant Isolates (*n*)			
	S ≤ ^4^	R > ^4^	*L. monocytogenes*	*L. innocua*	*L. seeligeri*	*L. newyorkensis*
PEN ^2^	0.125	0.125	8/8	8/8	1/1	5/5
ERY ^1^	1	1	0/8	0/8	0/1	5/5
Trim-sulfa ^1, 3^	0.06	0.06	0/8	2/8	0/1	0/5
GEN ^2^	2	2	1/8	1/8	0/1	0/5
TET ^2^	1	2	0/8	1/8	0/1	0/5
FOS ^2^	32	32	8/8	8/8	1/1	5/5
CIP ^2^	0.001	1	5/8	8/8	1/1	0/5
MOX ^2^	0.25	0.25	8/8	8/8	1/1	0/5

Seventeen isolates harbored AMR genes (Table 2), and five *L. newyorkensis* isolates showed no AMR genes. Five *L. newyorkensis* isolates were checked for phenotypic AMR to obtain more information on the antimicrobial susceptibility of this species. Breakpoints were evaluated using the EUCAST v 12.0 clinical breakpoints table [32]. PEN = benzylpenicillin; ERY = erythromycin; Trim-sulfa = trimethoprim-sulfamethoxazole; GEN = gentamicin; TET = tetracycline; FOS = fosfomycin; CIP = ciprofloxacin; MOX = moxifloxacin; ^1^ Minimal inhibitory concentration (MIC) breakpoints (mg/L) for *L. monocytogenes*; ^2^ MIC breakpoints (mg/L) for *Staphylococcus* spp.; ^3^ Trimethoprim–sulfamethoxazole in the ratio 1:19. The breakpoints are expressed as the trimethoprim concentration; ^4^ S = susceptible, standard dosing regimen; ^4^ R = resistant [39].

**Table 4:** Prevalence of virulence genes in 64 *Listeria* isolates.

	*prfA* % (*n*)	*hly* % (*n*)	*plcA* % (*n*)	*plcB* % (*n*)	*hpt* % (*n*)	*actA* % (*n*)	*inlA* % (*n*)	*inlB* % (*n*)	*mpl* % (*n*)
*L. monocytogenes*	100 (8/8)	100 (8/8)	100 (8/8)	100 (8/8)	100 (8/8)	100 (8/8)	100 (8/8)	100 (8/8)	100 (8/8)
*L. innocua*	0.00 (0/8)	0.00 (0/8)	0.00 (0/8)	0.00 (0/8)	0.00 (0/8)	0.00 (0/8)	0.00 (0/8)	0.00 (0/8)	0.00 (0/8)
*L. ivanovii*	66.67 (4/6)	66.67 (4/6)	0.00 (0/6)	0.00 (0/6)	100 (6/6)	0.00 (0/6)	66.67 (4/6)	66.67 (4/6)	0.00 (0/6)
*L. seeligeri*	91.67 (33/36)	91.67 (33/36)	91.67 (33/36)	91.67 (33/36)	100 (36/36)	0.00 (0/36)	0.00 (0/36)	0.00 (0/36)	91.67 (33/36)
*L. newyorkensis*	0.00 (0/5)	0.00 (0/5)	0.00 (0/5)	0.00 (0/5)	0.00 (0/5)	0.00 (0/5)	0.00 (0/5)	0.00 (0/5)	0.00 (0/5)
*L. grayi*	0.00 (0/1)	0.00 (0/1)	0.00 (0/1)	0.00 (0/1)	0.00 (0/1)	0.00 (0/1)	0.00 (0/1)	0.00 (0/1)	0.00 (0/1)

% (*n*) = percentage (number of isolates detected with virulence gene/total number of isolates of a designated species); *prfA* = positive regulatory factor A [44]; *hly* = listeriolysin O, LLO [45]; *plcA* = phosphatidylinositol-specific phospholipase C, Pl-PLC or PLC-A [45]; *plcB* = nonspecific phosphotidylcholine phospholipase C, PC-PLC or PLC-B [45]; *hpt* = hexose phosphates, HP [46]; *actA* = protein actA [45]; *inlA* = internalin A [45]; *inlB* = internalin B [45]; *mpl* = zinc metalloproteinase [47].

## Data Availability

The raw sequencing data were uploaded to the SRA of the NCBI database (https://www.ncbi.nlm.nih.gov/sra accessed on 28 February 2023) under the project number PRJNA935401.

## Supplementary Materials

The following supporting information can be downloaded at: https://www.mdpi.com/article/10.3390/foods12061120/s1, Figure S1: Dendrogram of *Listeria* isolates and their holding of origin based on the PIRATE v1.0.3 presence/absence analysis. Only one isolate of the species *L. grayi* (SWG4) was detected out of 123 samples of fresh and frozen soft fruit samples spread across supermarkets in the south of Bavaria, Germany. All other isolates were received from 39 FNAO-producing and -processing facilities. The holdings of origin are abbreviated randomly from A until the alphabetical letter T. The letters A, D, E, G, K, N, and O encode the processing companies (processing level), and the remaining letters stand for the primary production plants (farm and primary production level). The letter T represents a grocery. Table S1: MIC results of *Listeria* spp.
