# Supplementary material for: Genetic Characterization of Listeria from Food of Non-Animal Origin Products and from Producing and Processing Companies in Bavaria, Germany
Source: Foods. 2023 Mar 7;12(6):1120. doi: 10.3390/foods12061120 (PMC10048318; doi:10.3390/foods12061120)
Supplement: Supplementary file 1 [file foods-12-01120-s001.zip › Figure S1 Dendrogram of Listeria isolates.pdf]

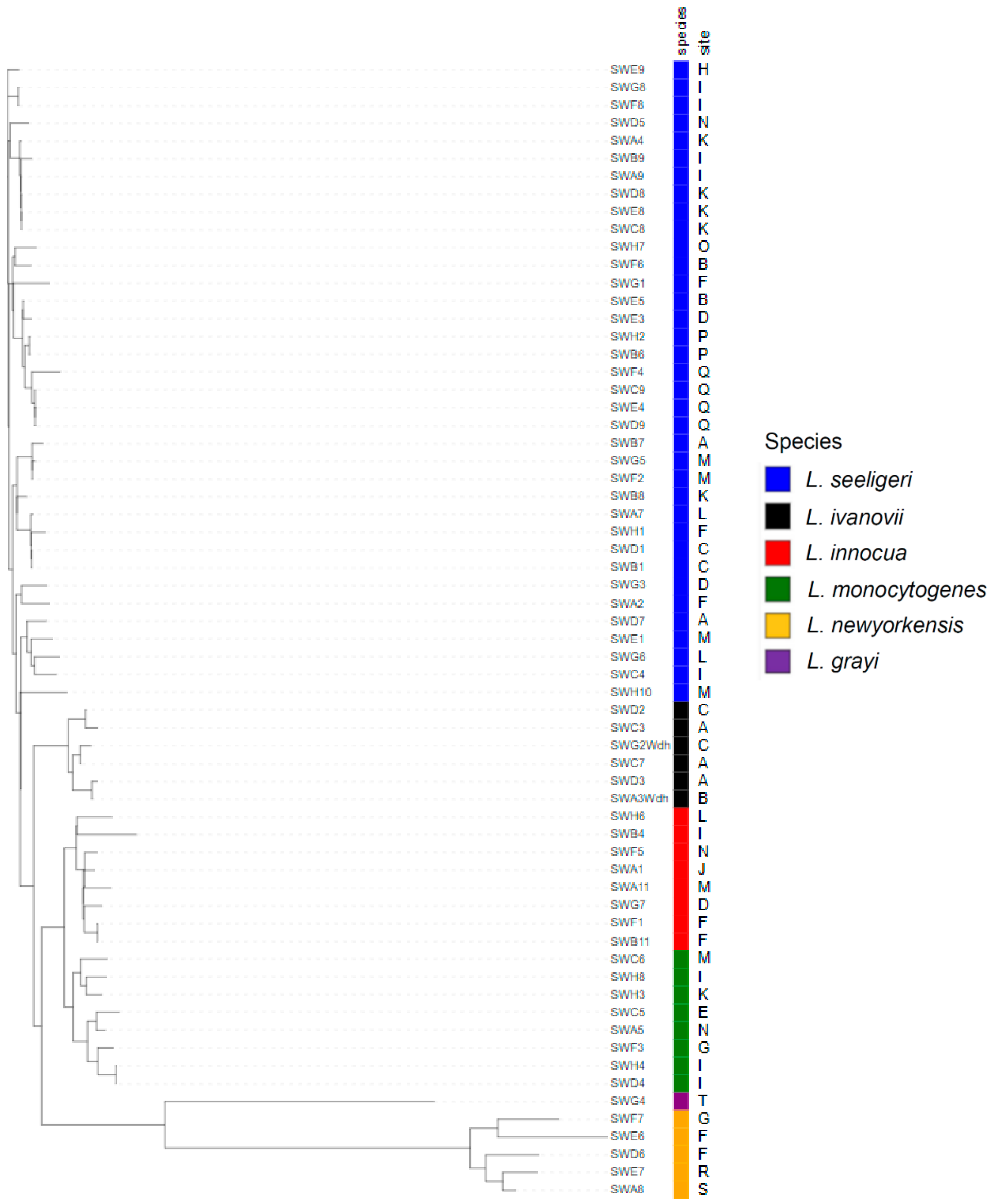

Figure S1. Dendrogram of *Listeria* isolates and their holding of origin based on the PIRATE v1.0.3 presence/absence analysis. Only one isolate of the species *L. grayi* (SWG4) was detected out of 123 samples of fresh and frozen soft fruit samples spread across supermarkets in the south of Bavaria, Germany. All other isolates were received from 39 FNAO-producing and processing facilities. The holdings of origin are abbreviated randomly from A until the alphabetical letter T. The letters A, D, E, G, K, N, and O encode processing companies (processing level), and the remaining letters stand for the primary production plants (farm and primary production level). The letter T represents a grocery.
